# Supplementary material for: One-hour coherent optical storage in an atomic frequency comb memory
Source: Nat Commun. 2021 Apr 22;12:2381. doi: 10.1038/s41467-021-22706-y (PMC8062444; doi:10.1038/s41467-021-22706-y)
Supplement: Supplementary file 1 — Supplementary Information [file 41467_2021_22706_MOESM1_ESM.pdf]

Supplementary Information for
One-hour coherent optical storage in an atomic frequency comb memory

Yu Ma,[1,2] You-Zhi Ma,[1,2] Zong-Quan Zhou,[1,2,*] Chuan-Feng Li,[1,2,†] and Guang-Can Guo[1,2]

[1] *CAS Key Laboratory of Quantum Information, University of Science and Technology of China, Hefei, 230026, P. R. China*

[2] *CAS Center For Excellence in Quantum Information and Quantum Physics, University of Science and Technology of China, Hefei, 230026, P. R. China*

Supplementary Note 1 - Pulsed Raman heterodyne detection

In our previous work [1], we use Raman heterodyne detection method to probe the continuous-wave nuclear magnetic resonance (NMR) of the ground state $^7F_0$ and excited state $^5D_0$ of $^{151}Eu^{3+}$:Y_2SiO_5. However, in this work, although the ground-state resonances can be detected using the same method, the excited-state resonances are difficult to detect due to the weaker radio-frequency (RF) fields generated by the Helmholtz coils.

To overcome this problem, we use a pulsed Raman heterodyne detection approach [2]. Optical pumping is employed to isolate a single class of ions in a specific ground-state level via class cleaning and spin polarization described in the paper. Then a probe pulse with a duration of 200 μs and a power of 4 mW resonant with the populated ground state and an arbitrary excited state is sent into the crystal, and at the mean time an RF pulse with a duration of 100 μs is applied to drive the hyperfine transitions in the excited state. The RF pulse is generated by a local oscillator with an amplitude of 0.6 V, and amplified by an amplifier with an output of 300 W to ensure a strong enough interaction with the excited-state hyperfine levels. The transmitted optical pulse is accompanied with a Raman scattered field if the RF pulse is resonant with the hyperfine transitions. A beat signal between the transmitted and scattered field is then captured by a photodetector and demodulated with a lock-in amplifier.

The resonance of the hyperfine transitions can be identified by varying the frequency of RF pulses. At least five resonances need to be found in order to determine the excited-state structure with six hyperfine levels. An example of the $|3\rangle_e \leftrightarrow |6\rangle_e$ transition at 124.52 MHz is shown in Supplementary Fig. 1. All the neighboring hyperfine transitions in the excited state are presented in Supplementary Table 1. As a comparison, we also provide the calculated results in the same field, based on the spin Hamiltonians determined in previous works [1, 3]. Note that due to the experimental errors, the calculated strength and direction of the ZEFOZ field is slightly different based on these two works. But we verify that the first-order Zeeman coefficient S_1 is the same, which ensures that the fields are physically equivalent. According to the data presented in Supplementary Table 1, the maximum error is larger than 1 MHz, which is the storage bandwidth of the memory. In addition, we note that the level structure of the $^7F_0$ ground state obtained here is significantly different from the results that reported in Ref. [4]. These results indicate the necessity of this experiment before determining the optical pumping strategies.

Supplementary Table 1. Experimental results of the transition frequency between the neighboring energy levels in the ZEFOZ field, as a comparison with the calculated results based on the fitted Hamiltonians determined in the previous work (calc. I and II refer to calculation based on [3] and [1], respectively). Unit: MHz.

Transition	Freq. (exp.)	Freq. (calc. I)	Freq. (calc. II)
$\|5\rangle_e \leftrightarrow \|6\rangle_e$	20.725	21.908	20.865
$\|4\rangle_e \leftrightarrow \|5\rangle_e$	79.903	79.899	79.856
$\|3\rangle_e \leftrightarrow \|4\rangle_e$	23.887	23.268	23.858
$\|2\rangle_e \leftrightarrow \|3\rangle_e$	56.199	55.999	56.089
$\|1\rangle_e \leftrightarrow \|2\rangle_e$	23.776	23.860	23.939

Supplementary Note 2 - Atomic frequency comb

The laser system at 580 nm is a specially-designed high-power laser. The seed laser is a semiconductor laser at 1160 nm (DL pro, Toptica) which is amplified to 30 W by a Raman fiber amplifier (PreciLasers). The laser at 580 nm is obtained by the single-pass second harmonic generation in a PPSLT crystal, with an output power of 6.2 W. The reference optical pulse for heterodyne detection is generated from another frequency-doubled semiconductor laser (TA-SHG, Toptica) with a locked frequency offset.

Before preparing the AFC in the $|3\rangle_g$ level, optical pumping sequence called class cleaning and spin polarization [5] is used to select one class of ions. The six pumping lights are resonant with $|1\rangle_g \leftrightarrow |1\rangle_e$, $|2\rangle_g \leftrightarrow |2\rangle_e$, $|3\rangle_g \leftrightarrow |3\rangle_e$, $|4\rangle_g \leftrightarrow |3\rangle_e$, $|5\rangle_g \leftrightarrow |3\rangle_e$, $|6\rangle_g \leftrightarrow |5\rangle_e$, respectively, with a power of 100 μW and a duration of 2 ms.

The structure of the AFC is presented in Supplementary Fig. 2a. We use a single-photon detector to record the absorption profile of the AFC prepared in $|3\rangle_g$. The

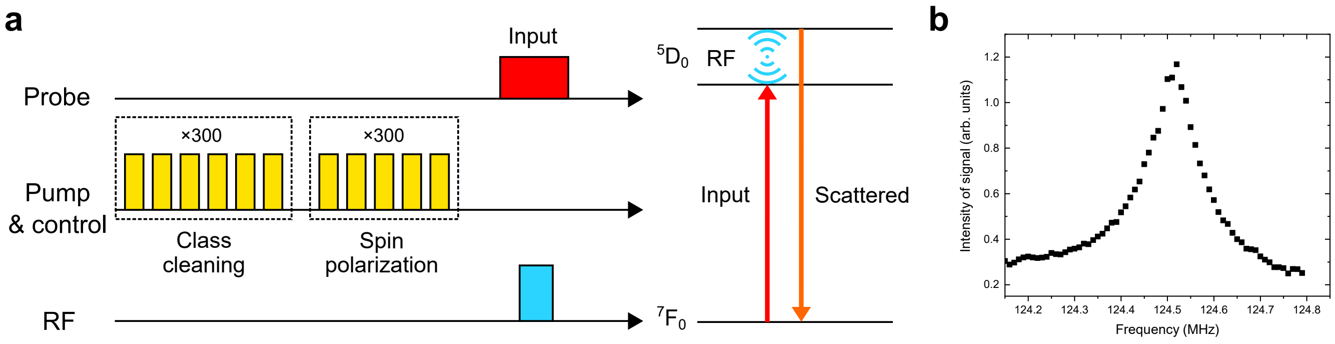

Supplementary Fig. 1. **Experimental sequence and results of the pulsed Raman heterodyne detection.** **a** The experimental sequence of the pulsed Raman heterodyne detection of the excited-state NMR in the ZEFOZ magnetic field. Class cleaning and spin polarization are employed to isolate a single class of ions in a specific ground-state energy level. The input pulse excites the ions to the excited state while an RF pulse simultaneously drives the excited-state hyperfine transitions. The scattered field is generated when the RF pulse is resonant with a specific excited-state transition. **b** Raman heterodyne detection of pulsed NMR of the $|3\rangle_e \leftrightarrow |6\rangle_e$ transition.

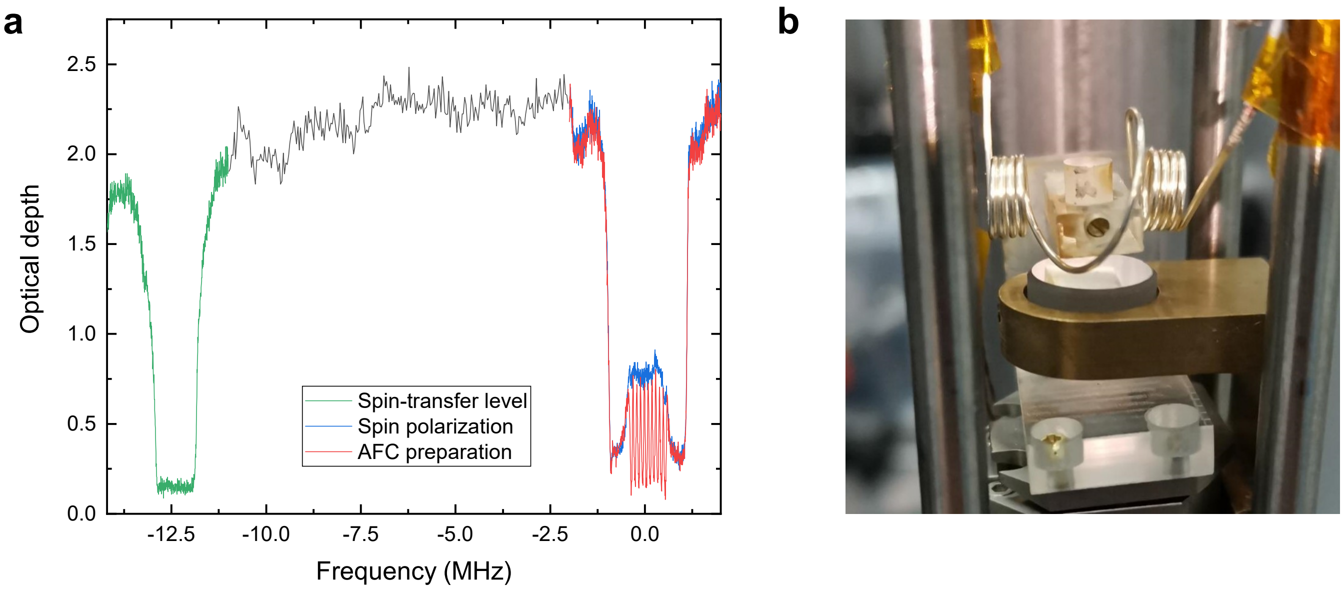

Supplementary Fig. 2. **AFC structure and a photo of the sample.** **a** The optical depth of the $|4\rangle_g \leftrightarrow |3\rangle_e$ (green) and $|3\rangle_g \leftrightarrow |3\rangle_e$ (blue) after the class cleaning and spin polarization, and the AFC structure with ten teeth prepared in the $|3\rangle_g$ (red). **b** The photo of the sample, which is fixed on a specially-designed holder mounted on the goniometers. The helmholtz coils are placed at the two sides of the sample and the mirror is below the sample. The external magnetic field and the incident beams are along the vertical direction.

AFC is prepared using a parallel method [6] within the 1 MHz bandwidth. The AFC has a comb periodicity of 100 kHz and a single peak width (FWHM) of $\gamma = 45$ kHz, leading to a finesse $F = 100/45 = 2.22$. The two-level AFC efficiency can be calculated as follows: [7]

$$\eta = \left(1 - e^{-(\alpha L/F)(\sqrt{\pi/4\ln 2})}\right)^2 e^{-\left(1/F^2\right)\left(\pi^2/2\ln 2\right)}, \quad (1)$$

where the optical depth after class cleaning and spin polarization is $\alpha L = 0.8$. The calculated efficiency η is 4.4%, which is close to our experimental result of 4.5%.

The control pulse used in the paper has a complex hyperbolic secant profile [8] to achieve efficient control over a large bandwidth. The transfer efficiency η_{control} of a single control pulse is estimated by comparing the spin-wave AFC echo with the two-level AFC echo.

In Supplementary Fig. 2b, we present a photo of the cylindrical sample and its surroundings. The optical surface is cut perpendicularly to the direction of the known ZEFOZ magnetic field in order to easily align the sample to the magnetic field of the magnet, which is approximately in the vertical direction.

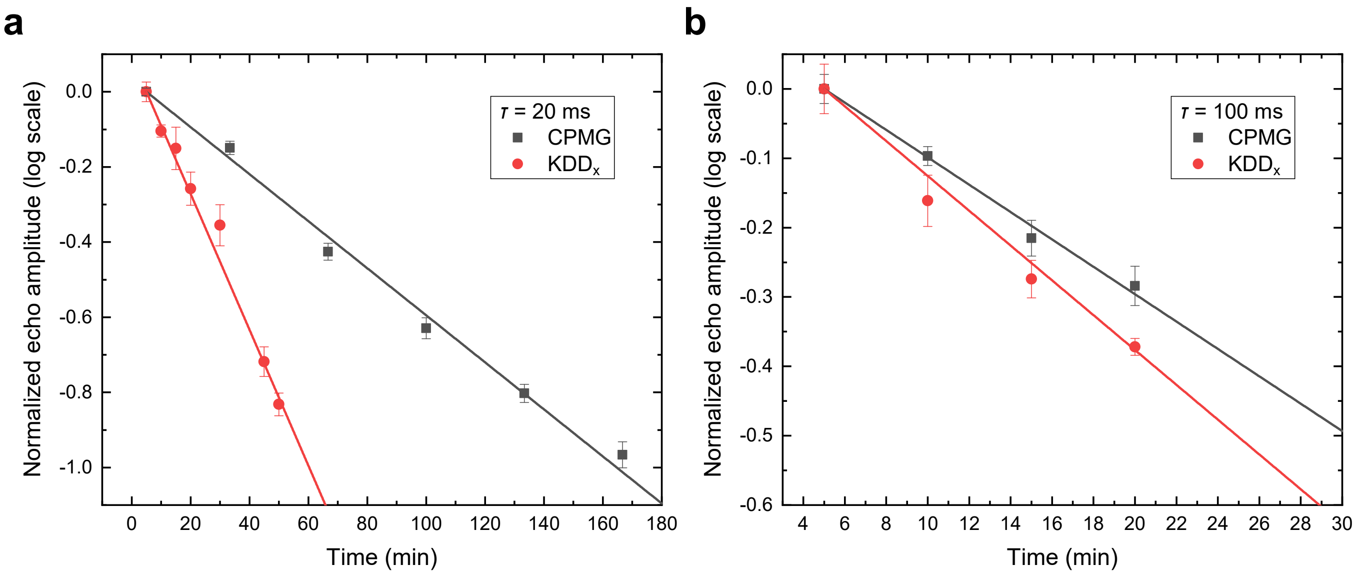

Supplementary Fig. 3. **Measurements of the spin coherence time in the ZEFOZ field.** **a** Decay of the spin echo with different dynamical decoupling sequences. CPMG (gray) has the longest coherence time of 2.68 ± 0.06 hours. KDD_x (red) has a coherence time of 0.95 ± 0.03. **b** When $\tau = 100$ ms, the coherence times are 50.6 ± 2.0 and 38.2 ± 2.0 minutes with CPMG and KDD_x, respectively. Each data point is averaged three times, and the error bars indicate one standard deviation of the echo area.

Supplementary Note 3 - Spin coherence time measurements

The width of the π pulse of 65.1 μs used in the dynamical decoupling and spin coherence time measurements is determined by implementing a spin nutation experiment [9]. The two-pulse phase memory time T_{M} [10] is determined to be 21.5 s, which is shorter than the 47 s in the previous work [4]. The possible reason is the inhomogeneity of the magnetic field, considering the longer sample used here.

In order to take advantage of the long spin coherence time of the $|3\rangle_{\mathrm{g}} \leftrightarrow |4\rangle_{\mathrm{g}}$ ($|-3/2\rangle_{\mathrm{g}} \leftrightarrow |+3/2\rangle_{\mathrm{g}}$) transition in the ZEFOZ field, we measure the coherence times by spin echo experiments with different dynamical decoupling sequences applied as shown in Supplementary Fig. 3. The spin echo signals are detected by Raman heterodyne detection. CPMG and KDD_x reach their longest coherence times of 2.68 ± 0.06 and 0.95 ± 0.03 hours respectively, when the interval τ between the π pulses is 20 ms. When τ is shorter than 20 ms, the heating of the Helmholtz coils and the infidelity of the π pulses will limit the spin coherence times. When $\tau = 100$ ms, the coherence times are 50.6 ± 2.0 and 38.2 ± 2.0 minutes with CPMG and KDD_x, respectively. The spin coherent lifetimes are essentially the same as the optical AFC storage lifetimes for $\tau = 100$ ms. We note that, due to strong fluctuations of the signal for optical storage with storage time shorter than 5 minutes, the lifetimes are fitted using the data starting from 5 minutes in Fig. 3 in the main text.

* zq_zhou@ustc.edu.cn
† cfli@ustc.edu.cn

[1] Ma, Y. *et al.* A Raman heterodyne study of the hyperfine interaction of the optically-excited state $^5D_0$ of Eu^{3+}:Y_2SiO_5. *J. Lumin.* **202**, 32–37 (2018).
[2] Mlynek, J., Wong, N. C., DeVoe, R. G., Kintzer, E. S. & Brewer, R. G. Raman heterodyne detection of nuclear magnetic resonance. *Phys. Rev. Lett.* **50**, 993–996 (1983).
[3] Cruzeiro, E. Z. *et al.* Characterization of the hyperfine interaction of the excited $^5D_0$ state of Eu^{3+}:Y_2SiO_5. *Phys. Rev. B* **97**, 094416 (2018).
[4] Zhong, M. *et al.* Optically addressable nuclear spins in a solid with a six-hour coherence time. *Nature* **517**, 177–180 (2015).
[5] Lauritzen, B. *et al.* Spectroscopic investigations of Eu^{3+}:Y_2SiO_5 for quantum memory applications. *Phys. Rev. B* **85**, 115111 (2012).
[6] Jobez, P. *et al.* Towards highly multimode optical quantum memory for quantum repeaters. *Phys. Rev. A* **93**, 032327 (2016).
[7] Afzelius, M., Simon, C., de Riedmatten, H. & Gisin, N. Multimode quantum memory based on atomic frequency combs. *Phys. Rev. A* **79**, 052329 (2009).
[8] Rippe, L., Nilsson, M., Kröll, S., Klieber, R. & Suter, D. Experimental demonstration of efficient and selective population transfer and qubit distillation in a rare-earth-metal-ion-doped crystal. *Phys. Rev. A* **71**, 062328 (2005).
[9] Ardelean, I., Kimmich, R. & Klemm, A. The nutation spin echo and its use for localized nmr. *J. Magn. Reson.* **146**, 43–48 (2000).
[10] Mims, W. B. Phase memory in electron spin echoes, lattice relaxation effects in $CaWO_4$:Er, Ce, Mn. *Phys.*

Rev. **168**, 370–389 (1968).
